# The Emerging Role of Flavonoids in Autism Spectrum Disorder: A Systematic Review

**DOI:** 10.3390/jcm12103520

**Published:** 2023-05-17

**Authors:** Rosa Savino, Alessandro Medoro, Sawan Ali, Giovanni Scapagnini, Michael Maes, Sergio Davinelli

**Affiliations:** 1 Department of Woman and Child, Neuropsychiatry for Child and Adolescent Unit, General Hospital “Riuniti” of Foggia, 71122 Foggia, Italy; 2 Department of Medicine and Health Sciences “V. Tiberio”, University of Molise, 86100 Campobasso, Italy; alessandro.medoro@unimol.it (A.M.); ali.sawani90@gmail.com (S.A.); giovanni.scapagnini@unimol.it (G.S.); 3 Department of Psychiatry, Faculty of Medicine, Chulalongkorn University, Bangkok 10330, Thailand; dr.michaelmaes@hotmail.com

**Keywords:** flavonoids, autism, nutrition, oxidative stress, inflammation

## Abstract

Although autism spectrum disorder (ASD) is a multifaceted neurodevelopmental syndrome, accumulating evidence indicates that oxidative stress and inflammation are common features of ASD. Flavonoids, one of the largest and best-investigated classes of plant-derived compounds, are known to exert antioxidant, anti-inflammatory, and neuroprotective effects. This review used a systematic search process to assess the available evidence on the effect of flavonoids on ASD. A comprehensive literature search was carried out in PubMed, Scopus, and Web of Science databases following the PRISMA guidelines. A total of 17 preclinical studies and 4 clinical investigations met our inclusion criteria and were included in the final review. Most findings from animal studies suggest that treatment with flavonoids improves oxidative stress parameters, reduces inflammatory mediators, and promotes pro-neurogenic effects. These studies also showed that flavonoids ameliorate the core symptoms of ASD, such as social deficits, repetitive behavior, learning and memory impairments, and motor coordination. However, there are no randomized placebo-controlled trials that support the clinical efficacy of flavonoids in ASD. We only found open-label studies and case reports/series, using only two flavonoids such as luteolin and quercetin. These preliminary clinical studies indicate that flavonoid administration may improve specific behavioral symptoms of ASD. Overall, this review is the first one to systematically report evidence for the putative beneficial effects of flavonoids on features of ASD. These promising preliminary results may provide the rationale for future randomized controlled trials aimed at confirming these outcomes.

## 1. Introduction

Autism spectrum disorder (ASD) is a complex neurodevelopmental disorder characterized by deficits in social interaction, impairments in language and communication abilities, and repetitive/stereotyped behaviors [1]. Emerging research suggests that the global prevalence of ASD has increased considerably over time, indicating a median prevalence of 100/10,000 within and across regions [2]. The vast majority of individuals with ASD do not receive an etiological diagnosis and receive a diagnosis of autism of unknown etiology. At this time, even after a thorough evaluation, the majority of cases of ASD have an unknown cause. Although there is no consensus on the causes of ASD, multiple risk factors have been proposed. Currently, the relationship between genetics and environment is thought to be a key driving force in the pathophysiology underlying ASD [3]. Large-scale genetic studies have identified hundreds of genes that have a role in synaptic development and function as risk factors for ASD pathogenesis. Genetic alterations associated with ASD may include different classes of genetic variants, such as single nucleotide polymorphisms (SNPs), mitochondrial DNA (mtDNA) variants, and structural variants (e.g., copy-number variations, inversions, large insertions, and tandem repeat expansions) [4,5]. Likewise, converging lines of evidence indicate that diverse prenatal, perinatal, and postnatal environmental exposures may induce epigenetic alterations in the brain. Environmental factors, such as maternal stress, infections, pollutants, poor diets, or overnutrition, can lead to altered methylation patterns in genes involved in neurodevelopment, immune function, detoxification pathways, and inflammatory responses [6,7].

A number of studies have also reported evidence that the pathogenesis of ASD is affected by mitochondrial dysfunction and oxidative stress [8]. Although there are several mechanisms leading to oxidative stress, the increased level of reactive oxygen species (ROS), resulting from mitochondrial dysfunction, contributes to generating an imbalance between the production of free radicals and the ability of the cells to detoxify their harmful effects. Importantly, the brain is particularly vulnerable to oxidative stress because of its higher oxygen consumption, higher lipid content, and weaker antioxidant defense. Oxidative damage to cellular macromolecules may cause a variety of physiological abnormalities in the brain, such as lipid peroxidation, protein and DNA oxidation, inflammation, and epigenetic dysregulation, which all may contribute to the clinical symptoms of ASD [9]. The presence of these alterations has been found not only in peripheral biomarkers but also in brain tissue derived from individuals diagnosed with ASD. Moreover, these abnormalities have been reported in different brain regions associated with speech and auditory processing, social behavior, memory, sensory, and motor coordination [8].

There is still no effective curative intervention for ASD, but new therapeutic models are focusing on the key role of oxidative stress as a potential target in ASD patients. Currently, pharmacological treatments can only be used to control symptoms related to ASD, including anxiety, hyperactivity, epilepsy, and obsessive behavior [10,11]. Likewise, psychosocial and educational interventions have had only limited success in reducing autistic symptomatology [12,13]. In this context, the emerging field of nutritional psychiatry may offer novel opportunities to prevent or manage psychiatric disorders, including ASD. Although epidemiological data are more widely available, an increasing amount of preclinical and intervention studies have started to describe the influence of food components and dietary patterns on mental outcomes associated with ASD [14,15,16]. It has become evident that nutrition affects early brain development and contributes to later mental health in youth with ASD. Indeed, many dietary agents are important for both the structural integrity of the brain and functional processes such as synaptogenesis and neurotransmitter synthesis [17]. Despite the lack of robust evidence to provide practical nutrition guidelines, several articles have suggested that specific dietary interventions may play a role in the management of some symptoms, functions, and clinical domains in patients with ASD [18,19]. Various medical nutrition approaches have been considered, including gluten-free, casein-free, and ketogenic diets, as well as probiotics, polyunsaturated fatty acids, camel milk, curcumin, and multivitamin and mineral supplements [20].

Flavonoids constitute a large group of polyphenolic compounds widely present in the human diet as they are found in fruits, vegetables, and plant-derived beverages. They are classified into six main subclasses, which include flavones, flavonols, flavanones, flavanols, anthocyanins, and isoflavones. These compounds have a wide range of favorable biochemical effects associated with various diseases, including neurodevelopmental and mood disorders [21,22,23]. Experimental studies have repeatedly demonstrated that flavonoids and their representative subclasses exert modulatory effects on biochemical signaling pathways associated with endogenous antioxidant systems, enhancement of mitochondrial functions, and inhibition of neuroinflammation. Recent evidence indicates that flavonoids play a role in regulating key signaling pathways, such as nuclear factor kappa-light-chain-enhancer of activated B cells (NF-κB) pathway, Janus kinase and signal transducer and activator of transcription proteins (JAK/STAT) pathway, Toll-like receptor (TLR) pathway, and cAMP response element-binding protein (CREB) pathway, involved in neuroinflammation associated with major neurological and psychiatric disorders, including ASD [24,25]. These effects may contribute to alleviating the pro-inflammatory state of children with ASD that exhibit heightened stress reactivity and hyperarousal symptoms. Furthermore, flavonoids may interact with a wide variety of neuronal signaling cascades, enhancing neuro-cognitive performance and increasing neurogenesis under healthy or pathological conditions [26]. Some natural flavonoids may also exert anxiolytic action through the activation of benzodiazepine receptors [27]. So far, the effect of flavonoids on ASD has not been systematically analyzed. Therefore, we aimed to systematically review all available findings generated from both preclinical and clinical studies investigating the role of flavonoids on ASD. 

## 2. Methods

This study followed the Preferred Reporting Items for Systematic Reviews and Meta Analyses (PRISMA) guidelines for systematic reviews [28].

### 2.1. Literature Search

We performed a comprehensive search on Scopus, PubMed, and Web of Science databases from their inception to December 2022 for preclinical studies and clinical investigations describing the effects of flavonoids on features of ASD. A systematic search was conducted using both free-text terms and controlled vocabulary. The search was performed using Boolean operators “AND” and “OR” to combine the following terms: “flavonoid” OR “flavonol” OR “flavanone” OR “flavone” OR “flavan-3-ol” OR “isoflavone” OR “daidzein” OR “genistein” OR “kaempferol” OR “apigenin” OR “catechin” OR “epicatechin” OR “epigallocatechin” OR “gallocatechin” OR “luteolin” OR “hesperetin” OR “quercetin” OR “biochanin” OR “theaflavin” OR “formononetin” OR “baicalein” OR “myricetin” OR “chrysin” OR “naringenin” OR “glycitein” OR “eriodictyol” OR “isorhamnetin” OR “thearubigin” OR “anthocyanin” OR “delphinidin” OR “peonidin” OR “malvidin” OR “anthocyanidin” OR “petunidin” OR “cyanidin” OR “pelargonidin” AND “autism” OR “autism spectrum disorder” OR “autistic”. At the same time, similar queries were respectively used for controlled vocabulary search: “flavonoids” [Mesh] AND “autism” [Mesh], INDEXTERMS “flavonoids” AND “autism”.

### 2.2. Eligibility Criteria and Data Extraction

We included preclinical and clinical studies that investigated the effect of flavonoid interventions on outcomes associated with ASD. Only reports that estimated the flavonoid content of foods or dietary supplements were included. We excluded articles for the following reasons: articles such as reviews, meta-analyses, conference papers, and book chapters and studies not published in English. The titles and abstracts obtained from the databases were independently reviewed by two authors (R.S. and S.A.). The full-text screening was conducted, excluding studies that did not meet the inclusion criteria. A third author (S.D.) was consulted in the case of disagreement about the eligibility of a study. In cases where full text was not available, we contacted the corresponding author and asked him to provide full-text publications within a 1-week time frame. The authors developed a data extraction form on an Excel sheet and the following data from eligible studies were extracted: author’s name; publication year; experimental model; study design; subject characteristics; intervention (duration, type of compounds, and dose); and results.

### 2.3. Risk of Bias

The risk of bias in the included preclinical studies was evaluated using the Systematic Review Centre for Laboratory Animal Experimentation (SYRCLE) tool [29]. This tool was developed to assess methodological quality and measure the bias in studies involving animal models. The SYRCLE tool considers the following domains: sequence generation, baseline characteristics, allocation concealment, random housing, investigator blinding, random outcome assessment, outcome assessor blinding, incomplete outcome data, and selective outcome reporting. These domains are related to five types of bias: selection bias; performance bias; detection bias; attrition bias; and reporting bias. For each included study, the bias types were classified as “high”, “low”, or “unclear”. Data on the housing conditions, such as light/dark cycle and temperature, were also extracted as an additional indicator of study quality.

## 3. Results

### 3.1. Selected Studies

**Figure 1:**
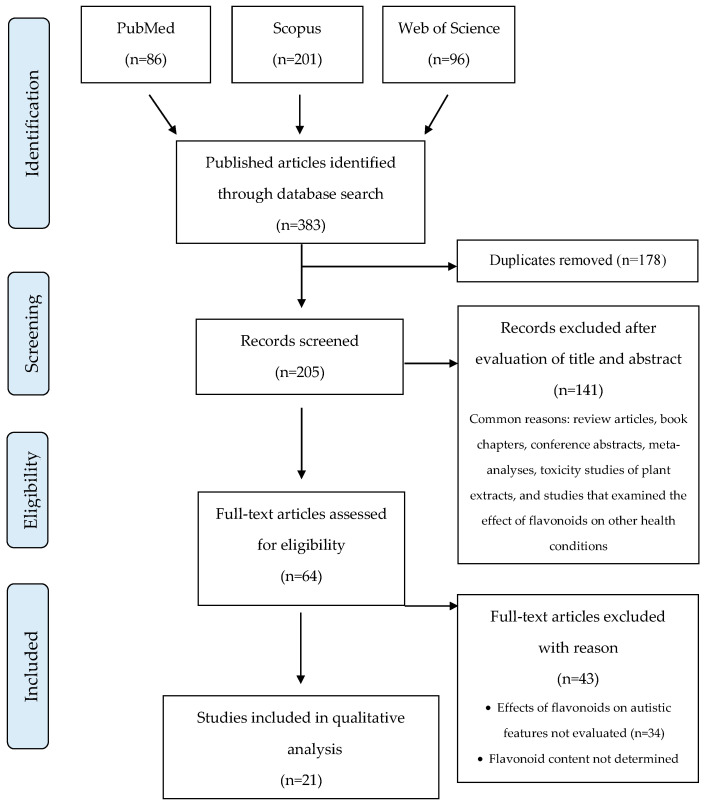
PRISMA flow diagram for systematic review.

As shown in Figure 1, we retrieved a total of 383 published studies from the three databases, but 178 were duplicates. We discarded 141 articles during the screening because they did not meet the inclusion criteria. We examined the remaining 64 articles for eligibility through full-text reading. Of these, 43 studies did not meet our inclusion criteria, or the full text was unavailable. Thus, a total of 21 studies were included in the final qualitative analysis. Seventeen papers specifically addressed the effects of flavonoids in animal models of ASD, 4 on ASD patients, and 1 study used both mice and humans as experimental systems. We present the main results of these studies in the following sections.

### 3.2. Preclinical Studies

The study characteristics and outcomes of the preclinical studies are summarized in Table 1. An extract of Bacopa monnieri, a nootropic herb, has been used to evaluate its neuroprotective effect in a valproic acid (VPA) model of ASD. The most abundant compound identified in this extract was luteolin, followed by apigenin. These flavonoids belong to the subclass of flavones. The results showed that B. monnieri extract, administered postnatally to rat pups at 80 mg/kg, may attenuate VPA-induced damage by restoring antioxidant enzymes and reducing inflammatory cytokines in the hippocampus and prefrontal cortex. In these brain regions, the treatment also reduced mRNA and protein expression of AMPA receptor, which plays a vital role in neurodevelopmental disorders such as ASD [30]. Furthermore, the in-silico analysis also showed a good binding profile of luteolin against the competitive antagonist binding site on the AMPA receptor. These effects were accompanied by improvements in learning and memory impairments, repetitive behavior, motor coordination, and social deficits [31].

Using a VPA model of autistic behaviors, the association of luteolin with palmitoylethanolamine (PEA) ameliorated autistic-like behavioral changes, including reduced sociability and increased anxiety-related behavior. The treatment not only reduced the expression of proinflammatory mediators, such as NF-kB, interleukin-1 beta (IL-1β), and tumor necrosis factor-alpha (TNF-α) but modulated apoptosis markers (Bax and Bcl-2) in hippocampus and cerebellum, also increasing neuroplasticity and neurogenesis in the hippocampus [32].

Naringenin, a flavonoid belonging to the flavanone subclass, has been reported to restore behavioral and biochemical deficits in a 3-4 months old male propanoic acid (PPA) rat model of ASD. Treatment was started on the 2nd day post-surgery and was continued till the 29th day. After the treatment period, naringenin encapsulated in polylactic-co-glycolic acid (PLGA) nanoparticles reduced the expression of matrix metalloproteinases-9 (MMP-9) and heat-shock proteins 70 (HSP-70). These proteins may have functions in driving the neuroinflammatory state associated with ASD [33]. In the same model, naringenin improved mitochondrial function in the brain by restoring, at least in part, the activities of mitochondrial enzyme complex I and II. Furthermore, naringenin nanoparticles reduced the expression of P-glycoprotein (P-gp), which is a transporter responsible for preventing the entry of various therapeutic moieties across the blood–brain barrier (BBB). These effects were also accompanied by improvements in sociability and perseverative behavior [33].

Quercetin, ubiquitous in plant-based foods and beverages, is categorized as flavanol. Using a VPA rat model, quercetin administered over 13 days (from the 6th to the 28th day of gestation) prevented alterations in social interaction and nociception in the rat pups. Likewise, treatment with quercetin prevented brain damage by improving oxidative stress parameters, mainly in the hippocampus and striatum [34].

Baicalin is a flavonoid of high biomedical value isolated from the root of Scutellaria baicalensis. Elesawy et al. demonstrated that postnatal treatment with baicalin might ameliorate neurochemical and behavioral alterations in a VPA rodent model of ASD. Specifically, baicalin improved neuronal mitochondrial functions, as demonstrated by increased synthesis of mitochondrial adenosine triphosphate (ATP) level and enhanced expression of mitofusin-2. This flavonoid elevated the level of sirtuin-1 (SIRT1) in the brain tissues and restored antioxidant enzymes such as superoxide dismutase (SOD) and catalase (CAT). Improvements in motor development, repetitive behavior, and social deficits have also been observed [35].

One study used a genetic loss-of-function model of an ASD risk gene (CNTNAP2) to conduct a pharmacological screen and identify novel compounds against ASD. The authors found that biochanin A, a phytoestrogenic isoflavone, might reverse the mutant behavioral phenotype in zebrafish larvae by decreasing night-time activity. Although biochanin A activated the expression of estrogen response genes, this transcriptional activation appeared to be independent of the behavioral rescue. In addition, early exposure to biochanin A did not reverse the GABAergic deficits in this model [36].

The 7,8-dihydroxyflavone (7,8-DHF) is a flavonoid that mimics the physiological actions of brain-derived neurotrophic factor (BDNF), activating tyrosine receptor kinase B (TrkB) and promoting neuronal survival, synpatogenesis, and axonal regeneration. Johnson et al. demonstrated that 7,8-DHF may attenuate some ASD symptoms in a Rett syndrome (RTT) mouse model. Oral administration of 7,8-DHF throughout life extended lifespan, increased the size of neuronal nuclei, and enhanced voluntary locomotor activity in these mice. In addition, 7,8-DHF partially ameliorated irregular breathing patterns and restored tidal volumes to wild-type levels [37]. In another study, 7,8-DHF reversed the altered synaptic structure and function caused by the genetic deletion of vaccinia-related kinase 3 (VRK3). VRK3 plays essential roles in synaptic structure and cognitive functions through the regulation of extracellular signal-regulated kinase (ERK), which is involved in the regulation of synaptic protein synthesis, dendritic morphology, and functional plasticity. Moreover, TrkB activation by 7,8-DHF treatment restored social interactions in VRK3-deficient mice showing autism-like behavior (12–15 weeks old) [38].

Genistein is a xenoestrogen (isoflavone) that may interfere with the development of estrogen-sensitive neural circuits and detrimentally affect the offspring microbiome–gut–brain axis. Many fetuses and infants are exposed to xenoestrogens through the placenta and milk. This exposure may increase the risk for ASD. One study exposed California mice offspring to bisphenol A (BPA), showing that these animals spent more time engaging in repetitive behaviors, which is considered a type of autistic-like behavior. Similarly, mice (90 days of age) exposed to genistein through the maternal diet engaged in similar repetitive behaviors and showed socio-communicative disturbances. These effects may be due to alterations in carbohydrate metabolism, phenylalanine, and tyrosine metabolism [39].

A study conducted by Khalaj et al. demonstrated that prenatal treatment with hesperetin, a flavonoid belonging to the flavanone subclass, may ameliorate autistic-like behaviors and oxi-inflammatory parameters (e.g., SOD, CAT, IL-6, and TNF-α) in the brain of rat pups exposed to VPA. Likewise, histopathological findings indicated that hesperetin protected Purkinje cells of the cerebellum [40]. Treatment with the flavanol catechin, a flavonoid derived from green tea, has been proven to target the nitric oxide pathway and ameliorate behavioral, biochemical, neurological, and molecular deficits in a PPA rat (3–4 months) model of ASD. Moreover, this compound improved the levels of neuroinflammatory and apoptotic markers such as TNF-α, IL-6, NF-κB, interferon-gamma (IFN-γ), HSP-70, and caspase-3 [41].

Alpha-glycosyl isoquercetin (AGIQ) is a flavonol glycoside that can be found in citrus fruits, red beans, and buckwheat. Continuous AGIQ treatment, starting during late gestation, ameliorated lipopolysaccharides (LPS)-induced pro-inflammatory responses and oxidative brain damage during infancy and prevented the expression of subsequent deficits in neurogenesis and behavior throughout the adult stage [42]. In another study, epigallocatechin gallate (EGCG), the most prevalent flavanol of green tea, alleviated neurological damage in a PPA 21-day-old rat model of ASD by increasing nerve growth factor (NGF), BDNF, TrkB, and calcium/calmodulin-dependent protein kinase II subunit alpha (CaMKII-α) levels and decreasing cAMP response element-binding protein (CREB) levels [43].

Prenatal prophylaxis with diosmin, a flavonoid structural analog of luteolin, showed to inhibit neuronal JAK2/STAT3 phosphorylation following the IL-6 challenge in a maternal immune activation (MIA) model of ASD. This flavone also improved behavioral deficits in social interaction in adult offspring [21].

Anthocyanins, a class of flavonoids present in berry fruits, are considered promising agents to reduce microglia-driven neuroinflammation. Serra et al. demonstrated that an anthocyanin-rich extract alleviated autism-like behaviors in a VPA-mouse model. At the same time, this extract decreased both neuroinflammation and gut inflammation, modulating the composition of the gut microbiota. Increased levels of serotonin and reduced synaptic dysfunction have also been demonstrated [44].

The Cdkl5 knockout (KO) mouse model is characterized by ASD features, intellectual disability, and early-onset epilepsy. The chronic administration of luteolin ameliorated hyperactive profile, memory ability, and motor stereotypies in this model. Moreover, this flavonoid also improved dendritic spine maturation and dendritic arborization of cortical neurons, increasing hippocampal neurogenesis [45]. In the same model, defective synaptic maturation in the hippocampi and cortices can be rescued through the intraperitoneal administration of EGCG, which is, however, not sufficient to normalize behavioral CDKL5-dependent deficits. Green tea flavonoid EGCG also restored defects in dendritic and synaptic development of primary Cdkl5 KO neurons [46].

### 3.3. Risk of Bias in Preclinical Studies

As shown in Table 2, most included preclinical studies (16 out of 17) described baseline characteristics. Nine studies showed a high risk of bias due to a lack of information on the technique for sequence generation, while seven studies were determined as an unclear risk since this information was not clearly stated. The allocation concealment was not described in detail in most of the studies (16 out of 17). An unclear risk of bias was determined for all studies due to ambiguity with random housing, but all studies clearly described housing conditions (e.g., light/dark cycle, temperature, and humidity). Data regarding investigator blinding was unclear in 11 studies. It was also unclear in most of the studies (14 out of 17) whether animals were selected at random for the outcome assessment. Blinding outcome assessment was reported in eight studies, while there was no clear evidence of blinding of the outcomes assessor in nine studies. The reporting of incomplete outcome data was unclear in 12 studies, while 4 studies included data collection from all outcome results. High risk was determined for one study due to missing outcome data. Sixteen studies were considered at low risk of bias from selective outcome reporting.

### 3.4. Clinical Studies

Five clinical studies involving a total of 145 children investigated the effects of flavonoids on ASD featuring (Table 3). However, one of these studies has already been presented in Section 3.2 since it used mice and humans as experimental systems. Similar to the results obtained from the mouse model, Bertolino et al. reported that a combined treatment of luteolin and PEA for 12 months improved the clinical picture in a 10-year-old male child with a reduction in stereotyped behaviors [32].

In a clinical trial of 17 children who had received glucocorticoids for at least 3 months to reduce neuroinflammation and improve autistic traits, supplementation with quercetin for 18 months ameliorated some features of ASD, such as deficits in sociability and impaired receptive language [47].

A 26-week, prospective, open-label trial demonstrated that a dietary supplement formulation containing luteolin and quercetin might provide significant benefits in ASD children both in adaptive functioning and behavioral difficulties. These flavonoids are considered safe, and the only adverse effect noted in the subjects was transient irritability [48]. Using the same flavonoids at the same dose, an uncontrolled open case series showed that treatment with luteolin and quercetin for 4 months might increase attention and sociability in children with ASD. The authors also reported an improvement in gastrointestinal dysfunction that may have had a substantial impact on the improvements seen in these children [49]. Likewise, an open-label trial on a cohort of 40 ASD children showed that the serum levels of IL-6 and TNF decreased significantly after a treatment period of 26 weeks with luteolin and quercetin, as compared with normotypic controls. This study also indicated a positive effect of luteolin and quercetin on the adaptive functioning of this cohort of ASD children [50].

## 4. Discussion

The scope of the present review was to systematically synthesize the current preclinical and clinical evidence on the effects of flavonoids in ASD and its associated symptoms. The majority of included studies found a positive result, suggesting that flavonoid administration may improve ASD features, including impairment in socialization and repetitive and stereotypic behaviors. Although all the included clinical studies found that flavonoids may help to mitigate the behavioral issues of ASD, there is a general paucity of randomized placebo-controlled trials evaluating the use of these compounds in children with ASD. Although placebo-controlled trials are considered the “gold standard” in medical research, the use of placebos in the pediatric field poses ethical and scientific challenges. First, children cannot exercise the principle of autonomy and are subject to parental decision-making on their behalf. Second, the placebo response might be larger in children/adolescents than in adults [51,52]. A recent study meta-analyzed the placebo response of core symptoms in pharmacological and dietary supplement ASD trials. Although no difference was found between age groups, these results should be interpreted with caution because the majority of studies were in pediatric populations. In order to increase the detection of the efficacy of experimental interventions for ASD, the same study also suggests considering the predictors of placebo response, such as the use of a threshold of core symptoms at inclusion, caregiver ratings, and flexible dosing. When large sample sizes and multiple sites are required, they should be carefully selected, trained, and monitored, trying to keep the number of sites at the minimum feasible [53]. In this review, examining only open-label studies and case reports/series, there is not sufficient evidence to support the clinical efficacy of flavonoids in ASD patients. Moreover, all the clinical studies had small sample sizes and used only two flavonoids such as luteolin and quercetin.

Luteolin is a common flavonoid present in many fruits, vegetables, and herbs. Although its bioavailability is low, luteolin represents one of the most powerful and effective flavonoids, which has displayed numerous biological properties, including anti-inflammatory, antioxidant, and neuroprotective properties [54]. Several studies indicate that the anti-inflammatory and antioxidant effects of luteolin are mediated through the inhibition of NF-kB and induction of redox-sensitive transcription factors involved in the activation of antioxidant defense systems [55,56]. Luteolin is also structurally related to 7,8-DHF, which was shown to have BDNF-like activity. In fact, it has been shown that luteolin may induce hippocampal neurogenesis by promoting the activation of BDNF [57]. A case report included in our review used luteolin in combination with PEA, an endocannabinoid-like lipid mediator with lipophilic nature. Many studies demonstrated that combined treatment with these compounds might stimulate both hippocampal neurogenesis and dendritic spine maturation to a greater extent than either luteolin or PEA alone [58,59]. Luteolin and quercetin share structural chemical features, and similar findings have been reported using quercetin. Despite some controversial results, quercetin increases survival against oxidative insults, providing neuroprotection through modulation of transcription factors and survival signaling cascades associated with antioxidant and anti-inflammatory pathways [60,61]. However, although delivery strategies are being developed (i.e., nanoformulations and lipid carriers), absorption and metabolic studies showed that quercetin and luteolin have very limited bioavailability [62,63]. Overall, the clinical utility of flavonoids, including luteolin and quercetin, to manage behavioral symptoms in patients with ASD remains to be validated by future clinical studies.

Due to the limited availability of postmortem brain tissue to determine the cellular and molecular alterations associated with ASD, animal models may help to investigate the neural structure of the autistic brain and define the neural systems that constitute the social brain and mediate repetitive behaviors. These preclinical models can also be employed to test the safety and effectiveness of potential therapeutic compounds [64]. Although animal models may have great translational value, the limitations of ASD models are rarely acknowledged, and the predictive validity of these models for humans is often overstated or misinterpreted. However, a recent literature review provided recommendations to identify limitations such as minimum sample sizes, sex controls, breeding schemas, housing conditions, genetic background, and task validation [65]. Despite this, a considerable number (16 out of 17) of the preclinical studies included in this review observed significant results of flavonoids against neurobehavioral alterations associated with ASD. Only one article reported a negative result, showing that genistein, a soy-derived isoflavone, may induce repetitive behavior and promote socio-communicative disturbances in California mice. These effects might be due to alterations in the microbiome–gut–brain axis induced by genistein exposure [39]. However, discordant results have been reported on the effects of genistein in animal behavior studies. Perinatal exposure of rats to genistein improved spatial learning and memory but impaired passive avoidance learning and memory [66]. Other studies with adult rodent models further suggest that genistein improves spatial and placement learning and memory [67,68]. Conversely, another study showed that male rats exposed to genistein through the maternal diet during both gestation and lactation exhibited spatial learning and memory deficits [69]. In humans, a recent national population-based observational cohort study examined the long-term neurodevelopmental outcomes during childhood following the consumption of soy formula rich in isoflavones during infancy. There was no evidence that soy formula increases the risk of epilepsy and ASD [70].

The preclinical studies included here used different animal models to investigate the effect of flavonoids on the behavioral and neurochemical characteristics of ASD. The VPA rodent model was the most frequently used model of ASD, followed by other experimental systems, such as the PPA rat model and genetic models. A good variety of flavonoids and their representative subclasses has been investigated in the research reports included in this review. One of the most examined flavonoid subclasses in the preclinical studies was flavones with a series of compounds such as luteolin, apigenin, baicalin, 7,8-DHF, and diosmin. Other flavonoid subclasses were flavanones (naringenin and hesperitin), flavonols (quercetin), isoflavones (genistein and biochanin A), flavanols (catechin and EGCG), and anthocyanins.

ASD neurobiology is thought to be associated with oxidative stress, as shown by increased levels of reactive oxygen and nitrogen species and alterations in other indicators of oxidative stress. ASD is also characterized by decreased glutathione reserve capacity. In particular, low levels of reduced glutathione (GSH), high levels of oxidized glutathione (GSSG), and alterations in the expressions of glutathione-related enzymes in the blood or brain appear to be important factors in the pathogenesis of ASD [9,71]. Findings from preclinical studies suggest that treatment with flavonoids, such as luteolin, quercetin, hesperetin, and catechin, can increase the activity of antioxidant enzymes, such as SOD, CAT, glutathione reductase (GRx), and peroxidase (GPx). Similarly, flavonoids may also reduce ROS and nitrite levels as well as decrease malondialdehyde MDA levels in ASD experimental models [31,34,40,41,42].

In recent years, the contribution of inflammation and neuro-immune dysregulations to ASD has been the object of intense research. Several studies have repeatedly found that increased systemic levels of pro-inflammatory mediators, altered patterns of immune cell response to activation stimuli, and abnormal microglia activation are hallmarks of ASD [72,73]. Consistent with their anti-inflammatory action, preclinical findings suggest that flavonoids, especially naringenin and hesperetin, can downregulate the expression of inflammatory mediators, such as IL-1β, IL-6, and TNF-α, through inhibition of NF-kB [31,32,33,40]. Isoquercitrin and anthocyanin may also reduce the reactivity of microglial activation markers, such as Iba1, CD68, and CD11, as well as astrocyte marker glial fibrillary acidic protein (GFAP) [42,44]. Some experimental studies also revealed that these effects are accompanied by improvement of mitochondrial function and gut microbiota composition. Interestingly, another possible mechanism, only partially explored in this range of studies, is that flavonoids, such as baicalin, naringenin, and anthocyanin, may modulate mitochondrial ATP production and factors involved in the respiratory chain deficiency [33,35,39,44]. Alongside an amelioration of the behavioral phenotype of ASD, findings from animal models seem to suggest a pro-neurogenic effect of flavonoids. Luteolin, EGCG, and isoquercitrin can activate the expression of neurotrophic factors, such as BDNF and nerve growth factor (NGF) [42,43,45]. The risk of bias in animal studies was evaluated according to the SYRCLE tool. The methodological quality of many studies was unclear since many items were unclear due to a lack of precise information. This suggests that there is much room for improvement. However, it is important to mention that the items in the SYRCLE tool are quite difficult to assess in animal intervention studies at present because protocols for animal studies are not yet registered in central, publicly accessible databases [29]. 

It is also important to note that the differences in bioavailability and absorption rates of the flavonoids are lacking in the included preclinical and clinical studies. The structure of flavonoids influences the rate of intestinal absorption, and several studies also suggest that the metabolites of flavonoids may be one of the characteristics responsible for their beneficial effects [74]. Therefore, given that flavonoid metabolism exhibits extensive variation between individuals, these aspects should be considered in future studies. Moreover, most of the preclinical studies examined here used different treatment durations and a large variety of flavonoid dosages. The dosages of flavonoids in animal studies are usually higher than those that are achievable by usual dietary intakes in humans.

The strength of this article is that it is the first review to systematically report evidence for the putative beneficial effects of flavonoids on ASD. Another strength of the current review is its broad scope and comprehensive search strategy, as we wanted to include a wide range of flavonoids and evaluate their effects on ASD features. However, this review has limitations which need to be acknowledged. Given the large heterogeneity among studies, no cumulative meta-analysis was conducted. This was due to the high heterogeneity among the studies with several experimental models used, distinct methodologies in the analyzed parameters, and high variation related to the dose and duration time of flavonoid supplementation. Another limitation of our review is that the risk of bias in the clinical investigations was not conducted due to the small number of the included studies and the lack of a reliable and adaptable tool to evaluate the methodological quality of these studies. However, the most important limitation of the present review is the lack of randomized, placebo-controlled trials, which prevents the strength of the conclusions that can be drawn from this review.

## 5. Conclusions

This systematic review summarizes the potential benefits of flavonoids on molecular and behavioral aspects of ASD. However, despite a variety of preclinical investigations that have been conducted, supporting, at least in part, the putative beneficial effects of flavonoids against ASD, blinded randomized clinical trials are needed. Thus, large-scale and well-designed controlled trials are essential to validate the preclinical findings and identify the most effective strategy (type of flavonoids, concentration, treatment duration) for patients with ASD.

## Figures and Tables

**Table 1:** Effects of flavonoids on ASD in preclinical studies.

Study	ASD Model	Flavonoid	Intervention Details	Key Findings
(Author, Year, and Reference)			(Duration and Dose)	
				
Abhishek et al.	VPA rat model	Flavonoid-rich extract	From PND 23 to 43	Increased levels of GSH, SOD, and CAT;
2022 [31]	(n = 24)	(leuteolin and apigenin)	20, 40, and 80 mg/kg	Decreased levels of MDA, IL-1β, IL- 6, and TNF-α;
				Reduced expression of AMPA receptor;
				Improved social deficits, repetitive behavior,
				and learning and memory impairments
				
Bertolino et al.	VPA mouse model	Luteolin with PEA	PND 15 for 3 months	Reduced levels of iNOS, GFAP, NF-kB, IL-1β,
2017 [32]	(n = 30)		1 mg/kg	TNF-α, and Bax;
				Increased expression of IkB-α and Bcl-2;
				Increased neurogenesis and synaptic plasticity;
				Improved social and nonsocial behaviors
				
Bhandari et al.	PPA rat model	Naringenin	From 2nd day post-	Decreased levels of TNF-α, MMP-9, and HSP-70;
2018 [33]	(n = 50)		surgery till 29th day	Reduced concentration of P-gp;
			25, 50 and 100 mg/kg	Improved mitochondrial complex activities;
				Ameliorated social interaction, sensorimotor
				dysfunction, and perseverative behavior
				
De Mattos et al.	Prenatal VPA rat model	Quercetin	From the 6th to the	Decreased levels of ROS, nitrites, and TBARS;
2020 [34]	(n = 12)		28th day of gestation	Increased concentration of SOD, CAT, GPx, and GST
			50 mg/kg	ALA-D;
				Reduced nociceptive response;
				Ameliorated social interaction
				
Elesawy et al.	VPA rat model	Baicalin	From PND 10 to 42	Increased levels of ATP, SIRT-1, mitofusin-2, GSH
2022 [35]	(n = 20)		100 mg/kg	SOD;
				Improved motor development, repetitive behavior,
				and social deficits
				
Hoffman et al.	Zebrafish larvae *CNTNAP2*	Biochanin A	From DPF 4 to 7	Reversed night-time hyperactivity;
2016 [36]	ASD model		0.1–1 μM	Increased expression of estrogen genes;
	(n = 302)			No effect GABAergic deficits
				
Johnson et al.	RTT mouse model	7,8-dihydroxyflavone	Throughout life	Extended lifespan;
2012 [37]	(n = 67)	(7,8 DHF)	80 mg/L	Improved growth (body mass);
				Increased the size of neuronal nuclei and
				voluntary wheel running;
				Improved breathing instability
				
Kang et al.	VRK3-deficient mice	7,8 DHF	3 days (CT)	Restored synaptic structure and function;
2017 [38]	(n > 35)		11 weeks (AT)	Improved social deficits and repetitive behavior;
			10 mg/kg	No effect on anxiety
				
Kaur et al.	Exposure to xenoestrogens	Genistein	Dams exposed to	Induced repetitive behavior;
2020 [39]	to induce ASD-like		genistein for 2 weeks	Promoted socio-communicative disturbances;
	behavior in California		prior to breeding	Increased number of metabolites involved in
	mice offspring		throughout gestation	carbohydrate metabolism and synthesis (females);
	(n = 40)		and lactation	Altered lysine degradation, phenylalanine and
			250 mg/kg	tyrosine metabolism, and urea cycle (males)
				
Khalaj et al.	VPA rat model	Hesperetin	From pregnancy to	Decreased levels of MDA;
2018 [40]	(n = 42)		PND 30	Increased activity of SOD, CAT, GPx, and GRx;
			10–20 mg/kg	Decreased level of IL-6 and TNF-α;
				Protected Purkinje cells of cerebellum;
				Improved social deficits and repetitive behavior
				
Mehta et al.	PPA rat model	Catechin	From 3rd day till	Reduced levels of MDA; homocysteine, nitrite; iNOS;
2021 [41]	(n = 60)		28th day	Improved levels of GSH, SOD, CAT;
			(3–4 months of age)	IL-6, IFN-γ, NF-kB, HSP-70, and caspase-3;
			50–100 mg/kg	Ameliorated sociability, repetitive behavior, and
				locomotor activity
				
Okano et al.	LPS rat model	Alpha-glycosyl isoquercitrin	From GD 1 to 18	Restored MDA levels and the GSSG/GSH ratio;
2022 [42]	(n = 71)	(AGIQ)	and from PND 0 to 77	Downregulation of IL-1α, IL-1β, and TNF- α;
			0.25–0.5 % in basal	Decreased populations of GFAP+ astrocytes;
			diet	Iba1+ microglia/macrophages, and CD68+;
				Upregulation of TGF-β1 and BDNF;
				Ameliorated fear memory acquisition, hippocampal
				neurogenesis and neuroinflammation
				
Ozdemir	PPA rat model	Epigallocatechin gallate	From day 5 to 35 in	Increased levels of NGF, BDNF, TrkB, and CaMKII-α
2020 [43]	(n = 28)	(EGCG)	21-day-old rats	Decreased levels of CREB;
			100 mg/kg	Improved spatial learning
				
Parker-Athill et al.	IL-6/MIA mouse model	Diosmin	Only once	Inhibited neuronal JAK2/STAT3 phosphorylation;
2009 [21]	(n = 22)		10 mg/kg	Reduced behavioural deficits in social interaction
				
Serra et al.	VPA mouse model	Anthocyanin-rich extract	From PND 30 to 55	Reduced IL-1β, TNF-α, IL-6, CD11b, and COX-2;
2022 [44]	(n = 16)		30 mg/kg	Increased Lactobacillales abundance;
				Decreased Clostridiales population;
				Promoted the production of serotonin;
				Decreased synaptic pruning dysregulation;
				Reduces social interaction deficits and repetitive
				behaviors
				
Tassinari et al.	Cdkl5 KO mouse model	Luteolin	From PND 90	Increased levels of BDNF and TrkB;
2022 [45]	(n = 57)		for 20 days	Improved neurogenesis, dendritic architecture, and
			10mg/kg	spine maturation;
				Ameliorated behavioural deficits
				
Trovò et al.	Cdkl5 KO mouse model	EGCG	From PND 60	Corrected synaptic defects;
2020 [46]	(n = 31)		for 30 days	Restored spine density and maturation;
			25 mg/kg	No effects on dendritic development and behavior
				

Autism spectrum disorder (ASD); Valproic acid (VPA); Postnatal day (PND); Glutathione (GSH); Superoxide dismutase (SOD); Catalase (CAT); Malondialdehyde (MDA); Interleukin-1 beta (IL-1β); Interleukin 6 (IL-6); Tumor necrosis factor alpha (TNF-α); α-amino-3-hydroxy-5-methyl-4-isoxazolepropionic acid (AMPA); Palmitoylethanolamide (PEA); Inducible nitric oxide synthase (iNOS); Glial fibrillary acidic protein (GFAP); Nuclear Factor-kB (NF-kB); Bcl-2-associated X protein (Bax); IkappaB kinase alpha (IkB-α); B-cell lymphoma 2 (Bcl-2); Propanoic acid (PPA); Matrix metalloproteinases-9 (MMP-9); Heat-shock proteins 70 (HSP-70); P-glycoprotein (P-gp); Reactive oxygen species (ROS); Thiobarbituric acid reactive substances (TBARS); Glutathione peroxidase (GPx); Glutathione-S-transferase (GST); aminolevulinic dehydratase (ALA-D); Adenosine triphosphate (ATP); sirtuin-1 (SIRT1); Contactin associated protein-like 2 (CNTNAP2); Days post fertilization (DPF); Rett syndrome (RTT); 7,8-dihydroxyflavone (7,8 DHF); Vaccinia-related kinase 3 (VRK3); Chronic treatment (CT); Acute treatment (AT); Glutathione reductase (GRx); Inducible nitric oxide synthase (iNOS); Interferon gamma (IFN-γ); Lipopolysaccharides (LPS); Gestational day (GD); Alpha-glycosyl isoquercitrin (AGIQ); Oxidized glutathione (GSSG); Interleukin 1 alpha (IL-1 α); Glial fibrillary acidic protein (GFAP+); Ionized calcium-binding adaptor protein-1 (Iba1+); Cluster of differentiation 68 (CD68+); Transforming growth factor-beta 1 (TGF-β1); Brain derived neurotrophic factor (BDNF); Epigallocatechin gallate (EGCG); Nerve growth factor (NGF); Tyrosine receptor kinase B (TrkB); Calcium/calmodulin-dependent protein kinase ii subunit alpha (CaMKII-α); cAMP response element-binding protein (CREB); Maternal immune activation (MIA); Janus tyrosine kinase-2/signal transducer and activator of transcription-3 (JAK2/STAT3); Cyclooxygenase-2 (COX-2); Knockout (KO).

**Table 2:** Risk of bias of included preclinical studies using the SYRCLE risk of bias tool.

Study (Author, Year, and Reference)	Selection Bias			Performance Bias		Detection Bias		Attrition Bias	Reporting Bias
	Sequence Generation	Baseline Characteristics	Allocation Concealment	Random Housing	Investigator Blinding	Random Outcome Assessment	Blinded Outcome Assessment	Incomplete Outcome Data	Selective Outcome Reporting
Abhishek et al., 2022 [31]	High	Low	High	Unclear	Unclear	Unclear	Unclear	Unclear	Low
Bertolino et al., 2017 [32]	High	Low	High	Unclear	Unclear	Unclear	Unclear	Unclear	Low
Bhandari et al., 2018 [33]	High	Low	High	Unclear	Unclear	Unclear	Unclear	Low	High
de Mattos et al., 2020 [34]	High	Low	High	Unclear	Unclear	Unclear	Unclear	Unclear	Low
Elesawy et al., 2022 [35]	Unclear	Low	Unclear	Unclear	Unclear	Unclear	Unclear	Low	Low
Hoffman et al., 2016 [36]	High	Unclear	High	Unclear	Unclear	Unclear	Low	Unclear	Low
Johnson et al., 2012 [37]	High	Low	High	Unclear	Low	Unclear	Low	Unclear	Low
Kang et al., 2017 [38]	High	Low	High	Unclear	Low	Unclear	Low	Unclear	Low
Kaur et al., 2020 [39]	Unclear	Low	Unclear	Unclear	Unclear	Low	Unclear	Unclear	Low
Khalaj et al., 2018 [40]	Unclear	Low	Unclear	Unclear	Unclear	Low	Unclear	Low	Low
Mehta et al., 2021 [41]	Low	Low	Low	Unclear	Low	Low	Low	Unclear	Low
Okano et al., 2022 [42]	Unclear	Low	Unclear	Unclear	Unclear	Unclear	Low	High	Low
Ozdemir 2020 [43]	Unclear	Low	Unclear	Unclear	Unclear	Unclear	Unclear	Low	Low
Parker-Athill et al., 2009 [21]	High	Low	High	Unclear	Unclear	Unclear	Unclear	Unclear	Low
Serra et al., 2022 [44]	High	Low	High	Unclear	Low	Unclear	Low	Unclear	Low
Tassinari et al., 2022 [45]	Unclear	Low	Unclear	Unclear	Low	Unclear	Low	Unclear	Low
Trovò et al., 2020 [46]	Unclear	Low	Unclear	Unclear	Low	Unclear	Low	Unclear	Low

**Table 3:** Effects of flavonoids on ASD in clinical studies.

Study	Study Design	Study Population	Flavonoids	Intervention Details	Key Findings
(Author, Year, and Reference)				(Duration and Dose)	
					
Bertolino et al.	Case report	A 10-year old	Luteolin with PEA	12 months	Improved sociability and motor
2017 [32]		male child with ASD		700 mg + 70 mg b.i.d.	stereotypies;
					Reduced enuresis
					
Ekici	Open-label study	17 children (4-8 years old)	Quercitin	18 months	Some improvement in social interaction,
2020 [47]		(n = 14 boys; n = 3 girls)		250 mg/day	language skills, and EEG
					
Taliou et al.	Open-label study	50 children (4–10 years old)	Luteolin and quercitin	26 weeks	Improved adaptive functioning;
2013 [48]		(n = 42 boys; n = 8 girls)		200 mg/day	Ameliorated behavioral difficulties;
					Transient irritability
					
Theoharides et al.	Case series	37 children (4–14 years old)	Luteolin and quercitin	4 months	Improved gastrointestinal dysfunction
2012 [49]		(n = 29 boys; n = 8 girls)		200 mg/day	and allergy;
					Increased eye contact, attention
					and social interaction;
					Good tolerability
					
Tsilioni et al.	Open-label study	40 children (4–10 years old)	Luteolin and quercitin	26 weeks	Decreased IL-6 and TNF;
2015 [50]		(n = 34 boys; n = 6 girls)		200 mg/day	No effect on CRH and NT;
					Improved sociability, communication
					daily living skills
					

Autism spectrum disorder (ASD); Palmitoylethanolamide (PEA); Electroencephalography (EEG); Interleukin-6 (IL-6); Tumor necrosis factor (TNF); Corticotropin-releasing hormone (CRH); Neurotensin (NT).
